# Thoracic Aortic Aneurysm Following Blunt Trauma in a Patient with a Monoallelic *SLC2A10* Variant: A Case Report

**DOI:** 10.3400/avd.cr.25-00135

**Published:** 2026-03-01

**Authors:** Satoshi Uesugi, Naoyuki Kimura, Shogo Saito, Mamoru Arakawa, Arata Muraoka, Yasushi Imai, Koji Kawahito

**Affiliations:** 1 Division of Cardiovascular Surgery, Department of Surgery, Jichi Medical University, Shimotsuke, Tochigi, Japan; 2 Division of Cardiovascular Medicine, Department of Internal Medicine, Jichi Medical University, Shimotsuke, Tochigi, Japan; 3 Division of Clinical Pharmacology, Department of Pharmacology, Jichi Medical University, Shimotsuke, Tochigi, Japan

**Keywords:** arterial tortuosity syndrome, *SLC2A10* gene, thoracic aortic aneurysm

## Abstract

A female in her early 40s with no skeletal abnormalities was incidentally found to have a 45-mm saccular aneurysm at the aortic isthmus during evaluation for pharyngitis. She had sustained blunt trauma 20 years earlier, resulting in multiple fractures and pneumothorax. Her family history included premature vascular or sudden death and scoliosis. Imaging showed no arterial tortuosity. She underwent successful open surgical repair. Histologic examination revealed disorganized elastic fibers with irregular thickening and partial loss of lamellar architecture. Postoperative genetic testing identified a heterozygous missense variant in *SLC2A10*, suggesting a possible association between monoallelic variants and vascular fragility.

## Introduction

Arterial tortuosity syndrome (ATS) is a rare autosomal recessive connective tissue disorder resulting from loss-of-function mutations in both alleles of the *SLC2A10* gene.^1)^ ATS is characterized by widespread tortuosity of large- and medium-sized arteries, predisposing affected individuals to aneurysm formation, arterial dissection, and ischemic events.^1–3)^ While a definitive diagnosis of ATS typically requires biallelic pathogenic variants, recent advances in genetic screening technology have led to occasional identification of heterozygous variants in patients with incomplete or atypical ATS phenotypes. The clinical significance of such monoallelic *SLC2A10* variants remains undetermined; however, a previous report has suggested that they may be associated with increased vascular fragility in selected individuals.^4)^ Herein, we report a traumatic thoracic aortic aneurysm in a patient harboring a heterozygous *SLC2A10* variant, without radiological features of arterial tortuosity or elongation, but with a notable family history suggestive of an underlying heritable connective tissue disorder.

## Case Report

**Fig. 1:**
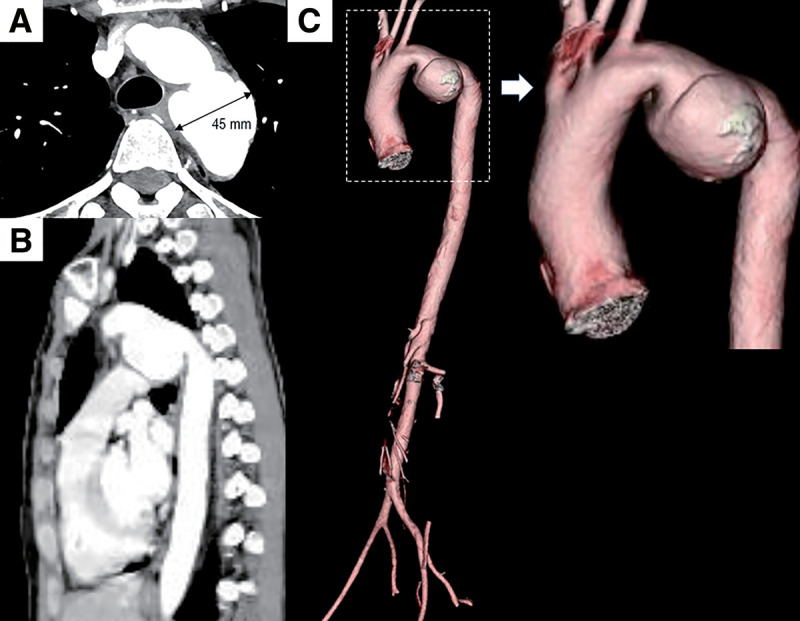
Preoperative computed tomography images. Axial (**A**), sagittal (**B**), and 3-dimensional reconstruction (**C**) views reveal a saccular aneurysm in the proximal descending thoracic aorta, without evidence of tortuosity in the thoracic or abdominal aorta.

A female in her early 40s presented to a local clinic with a sore throat and fever and was referred to our emergency department for further evaluation. She was diagnosed with acute pharyngitis, and her symptoms improved with antibiotic therapy. During screening chest computed tomography (CT), a 45-mm saccular aneurysm of the aortic isthmus was discovered incidentally, without evidence of tortuosity in the thoracic or abdominal aorta (**Fig. 1**). Thus, the patient was referred to our cardiovascular surgery department for further assessment and treatment.

Her past medical history was notable for a motor vehicle accident approximately 20 years earlier, resulting in blunt chest trauma with left pneumothorax and multiple fractures, including pelvic, mandibular, and left lower leg fractures. Two years before her referral to our department, she had been diagnosed with hypertension, but no antihypertensive treatment had been initiated. Her family history was notable in that her father had died of cerebral hemorrhage at age 31. Of his 4 siblings, 2 had died before age 30. The patient’s younger brother had undergone surgery for sigmoid colon perforation. Of the patient’s 2 children, her elder son had scoliosis, whereas her younger son had no apparent skeletal abnormalities.

**Fig. 2:**
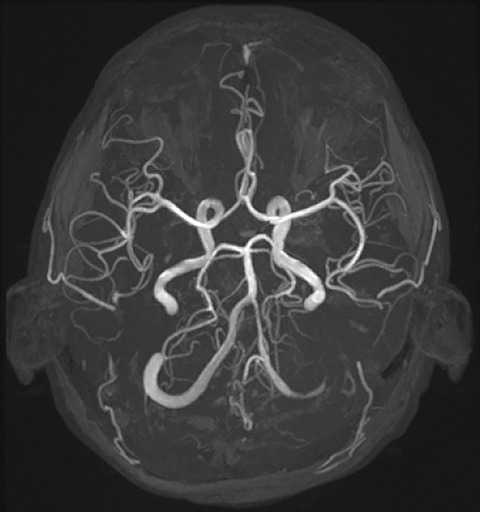
Preoperative magnetic resonance angiography showing no evidence of intracranial artery tortuosity or aneurysm formation.

The patient herself had no apparent skeletal abnormalities. On ophthalmologic examination, there were no obvious abnormalities, including lens dislocation. Preoperative magnetic resonance imaging of the brain revealed no intracranial abnormalities (**Fig. 2**).

**Fig. 3:**
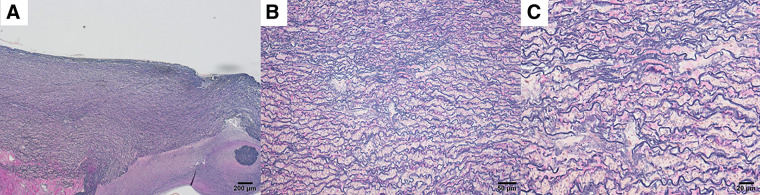
Histologic features of the traumatic thoracic aortic aneurysm (Elastica–Van Gieson stain). (**A**) At low power, the aortic wall shows loss of normal medial lamellar organization. (**B**) At intermediate magnification, elastic lamellae appear irregular and thickened. (**C**) At high power, elastic fibers appear fragmented with reduced lamellar integrity and expansion of the interlamellar matrix.

The patient underwent open surgical repair of the saccular aneurysm in the proximal descending thoracic aorta via left thoracotomy under cardiopulmonary bypass with circulatory arrest. Intraoperatively, extensive adhesions between the left lung and chest wall were noted, suggestive of prior trauma. Open proximal anastomosis was performed using a 20-mm Hemashield graft (Getinge, Gothenburg, Sweden). Operation time was 570 min, cardiopulmonary bypass time was 183 min, and the blood loss volume was 2205 mL. The postoperative course was uneventful, with the patient extubated on postoperative day 1 and discharged home on day 17. Postoperative CT showed no graft abnormalities. Histologic examination of the resected aorta revealed disorganized and fragmented elastic fibers with irregular thickening and partial loss of its lamellar architecture (**Fig. 3**). All histological analyses were performed on tissue obtained from the aneurysmal portion of the descending aorta. Pathological features characteristic of a traumatic pseudoaneurysm, such as complete disruption of the medial layer or fibrous replacement of the aortic wall, were not observed in this case.

After hospital discharge, the patient requested genetic testing for aortic disease, given the prevalence of early-onset disease and premature deaths among family members and the presence of thoracic skeletal deformities in her child. With informed consent, a blood sample was submitted to the Kazusa DNA Research Institute (Chiba, Japan) for next-generation sequencing (NGS)-based gene panel analysis targeting heritable aortic disorders. Genomic DNA extracted from peripheral blood was subjected to hybridization-based target enrichment and short-read NGS, focusing on the protein-coding exons and splice-site junctions in 18 genes, including *SLC2A10*, associated with heritable aortic disease. Variants with allele frequencies below 0.1% were specifically reported. Copy number variations and large structural variants were not assessed due to technical limitations, and somatic mosaicism was reported only when detectable by variant callers. According to the report from the Kazusa DNA Research Institute, a heterozygous rare variant in *SLC2A10* (c.692G>A, p.Arg231Gln) was identified, reported as a rare variant (allele frequency 0.000020) with conflicting classifications of pathogenicity. The variant has been classified with conflicting interpretations of pathogenicity. No second pathogenic variant or evidence of mosaicism was detected.

## Discussion

ATS is a connective tissue disorder resulting from a loss-of-function mutation in the *SLC2A10* gene encoding the facilitative glucose transporter GLUT10 protein.^1,3)^ Mutations affecting this transporter impair the production of collagen and elastin, resulting in arterial fragility and enhanced activation of the transforming growth factor-β signaling pathway.^5)^ The prevalence of ATS is quite low, with around 100 patients with ATS having been reported in the literature to date.^2)^ Clinical presentations of ATS include characteristic craniofacial features and connective tissue abnormalities, such as hyperextensible skin, cutis laxa, diaphragmatic hernia, and Marfanoid skeletal features.^1,6,7)^ Notably, scoliosis, which was observed in the patient’s older child, has been reported in 27% (12/44) of individuals diagnosed with ATS.^1)^ Tortuosity of the aorta and/or mid-sized arteries is invariably present.^1,7)^ A review by Wessels et al. indicated a poor prognosis in ATS patients, with mortality rates reaching up to 40% before the age of 5.^6)^ The exact causes of death remain unclear; however, respiratory failure, pulmonary infection, and organ infarction may contribute to early death in cases of ATS.^1,8)^ Recent studies have reported more favorable prognoses,^1,7)^ but the natural history, including aortic event rates and overall outcomes, remains incompletely understood.

Our case was that of a young-onset aortic isthmus aneurysm in the setting of previous systemic trauma. The impact of the multiple traumatic injuries was confirmed intraoperatively by extensive adhesions between the lung and chest wall. Considering these factors, the patient was diagnosed with a traumatic thoracic aortic aneurysm. Blunt traumatic thoracic aortic injury, the second most common cause of death in trauma patients, is caused by high deceleration forces applied to the aorta.^9)^ Recent findings suggest that traumatic aortic injury, though caused by external trauma, may also involve genetic predispositions, for example, to anatomical abnormalities of the aorta.^10)^

In the case reported herein, a monoallelic missense variant in the *SLC2A10* gene was identified in a patient who showed no evidence of tortuosity in the cerebral vessels, thoracoabdominal aorta, or medium-sized arteries. This observation aligns with a previous report by Callewaert et al., in which none of the 8 individuals carrying monoallelic *SLC2A10* variants exhibited vascular anomalies.^7)^ Although such variants have traditionally been considered asymptomatic, a study by Jiang et al. revealed an increased risk of vascular disease, including peripheral arterial disease.^4)^ In addition, a recent case report described a young adult carrying a heterozygous *SLC2A10* variant who developed a significant aortic root aneurysm requiring surgical repair, suggesting that monoallelic variants may also contribute to thoracic aortic aneurysm formation.^11)^ Histologic abnormalities associated with ATS were detailed by Beyens et al.,^1)^ who showed, using Verhoeff–Van Gieson staining, reduced smooth elastic lamellae and highly disorganized, thickened, and fragmented elastic fibers, most pronounced in the aortic wall. In our patient, despite the heterozygous *SLC2A10* mutation, histologic examination likewise revealed fragmented and irregular elastic fibers with partial loss of lamellar architecture, findings in accord with the vascular pathology reported in ATS. This heterozygous variant is located in a highly conserved region of the gene, suggesting possible functional significance despite its monoallelic status. Taken together, these findings indicate that the monoallelic *SLC2A10* variant may have contributed to the development of our patient’s traumatic thoracic aortic aneurysm. Finally, the presence of scoliosis in one of the patient’s sons underscores the importance of genetic counseling and careful clinical follow-up of family members, as monoallelic *SLC2A10* variants may be associated with variable and incomplete phenotypic expression.

## Conclusion

Findings in this case suggest a potential association between monoallelic *SLC2A10* variants and vascular fragility, even in the absence of arterial tortuosity. While the described thoracic aortic aneurysm was likely trauma-related, our patient’s genetic background and family history point to a possible underlying predisposition. The clinical significance of heterozygous variants in connective tissue genes warrants further investigation, which we anticipate as genetic testing becomes more widely available.
